# Systemic Inflammation and the Brain: Novel Roles of Genetic, Molecular, and Environmental Cues as Drivers of Neurodegeneration

**DOI:** 10.3389/fncel.2015.00028

**Published:** 2015-02-02

**Authors:** Roman Sankowski, Simone Mader, Sergio Iván Valdés-Ferrer

**Affiliations:** ^1^Elmezzi Graduate School of Molecular Medicine, Manhasset, NY, USA; ^2^Feinstein Institute for Medical Research, Manhasset, NY, USA; ^3^Department of Neurology, Instituto Nacional de Ciencias Médicas y Nutrición Salvador Zubirán, México City, Mexico

**Keywords:** neurodegeneration, systemic inflammation and sepsis, autoimmune disorders, anti-brain antibodies, TNF, HMGB1, connectome

## Abstract

The nervous and immune systems have evolved in parallel from the early bilaterians, in which innate immunity and a central nervous system (CNS) coexisted for the first time, to jawed vertebrates and the appearance of adaptive immunity. The CNS feeds from, and integrates efferent signals in response to, somatic and autonomic sensory information. The CNS receives input also from the periphery about inflammation and infection. Cytokines, chemokines, and damage-associated soluble mediators of systemic inflammation can also gain access to the CNS via blood flow. In response to systemic inflammation, those soluble mediators can access directly through the circumventricular organs, as well as open the blood–brain barrier. The resulting translocation of inflammatory mediators can interfere with neuronal and glial well-being, leading to a break of balance in brain homeostasis. This in turn results in cognitive and behavioral manifestations commonly present during acute infections – including anorexia, malaise, depression, and decreased physical activity – collectively known as the sickness behavior (SB). While SB manifestations are transient and self-limited, under states of persistent systemic inflammatory response the cognitive and behavioral changes can become permanent. For example, cognitive decline is almost universal in sepsis survivors, and a common finding in patients with systemic lupus erythematosus. Here, we review recent genetic evidence suggesting an association between neurodegenerative disorders and persistent immune activation; clinical and experimental evidence indicating previously unidentified immune-mediated pathways of neurodegeneration; and novel immunomodulatory targets and their potential relevance for neurodegenerative disorders.

## Introduction: An Evolutionary Perspective

All multicellular organisms have germ-line encoded surveillance systems destined to detect potentially dangerous, perhaps lethal, invaders (Medzhitov and Janeway, 1997). This extremely effective response, the innate immunity, depends on detection of specific molecular patterns present in invaders, but not expressed by self-tissues. Innate immunity aroused approximately 600 million years ago, and is evolutionarily conserved across species and kingdoms. The main components of innate immunity – from toll-like receptors (TLR) to inflammasomes – are highly conserved from plants to mammals (Jones and Dangl, 2006). Adaptive immunity appeared much later in evolution, perhaps 500 million years ago, and is only present in vertebrates (Cooper and Alder, 2006; Moresco et al., 2011). Unlike innate immunity, adaptive immunity depends on one individual’s experience, in which adaptive responses form with high efficiency only against antigens that have been recognized and presented by the innate immunity. Although the origin of the nervous system remains controversial, the first centralized nervous system probably appeared with the first bilateral organisms (organisms with two relatively identical halves, called *bilaterians*) during the Ediacaran period around 550 million years ago (Knoll et al., 2004). Ever since, both nervous and immune systems have co-evolved and are in constant communication.

The nervous system of the evolutionarily ancient nematode *Caenorhabditis elegans* has the ability to regulate innate immune responses (Andersson and Tracey, 2012), and aid in decision-making regarding finding bacteria that can be used as food and avoiding pathogenic bacteria (Reddy et al., 2009). In mammals, the nervous system also has the ability to sense inflammatory stimuli directly, thus allowing to recognize a potential source of damage through generation of pain, and to modulate the response to infection (Mina-Osorio et al., 2012; Chiu et al., 2013a). Although the afferent pathways and integration of immune information in the brain are areas of active research, there is evidence that central muscarinic signaling modulates inflammation in experimental sepsis (Pavlov et al., 2009; Rosas-Ballina et al., 2015), obesity (Satapathy et al., 2011), and inflammatory colitis (Ji et al., 2014). The efferent axis of neuroimmune control is better understood after the cholinergic anti-inflammatory pathway (CAP) (Borovikova et al., 2000), a cholinergic reflex system that regulates inflammation via the vagus nerve that stimulates the splenic nerve to release noradrenaline. Noradrenaline in turn stimulates a subset of acetylcholine (ACh)-producing splenic T-cells (CD4^+^CD44^hi^CD62L^lo^) to release ACh, which binds to α7 nichotinic receptors on the surface of macrophages, resulting in down-regulation of TNF by blocking the nuclear translocation of nuclear factor kappa B (NF-κB) (Rosas-Ballina et al., 2011). Thus far, this is a unique scenario in which an immune cell acts as interneuron in a reflex system. Electrical as well as chemical stimulation of the CAP have been shown to decrease the inflammatory burden and increase survival of experimental sepsis (Borovikova et al., 2000; Bernik et al., 2002).

Neuroimmune modulation is a flexible phenomenon that relies on environmental cues from a continuously changing milieu. People undergoing persistent stress have been found to display abnormal immune responses. For instance, caregivers of Alzheimer disease (AD) patients have higher levels of anxiety and depression than age-matched controls; otherwise, these healthy caregivers also have reduced total T-cells and T helper cells in peripheral circulation, as well as higher titers of anti-Epstein–Barr virus antibodies (Kiecolt-Glaser et al., 1987). Moreover, the observed immune and behavioral changes are increased in proportion to disease progression. T-cell response to mitogenic stimuli progressively decreases, and while all subjects have the same number of infectious episodes, the number of days unable to perform activities of daily living, as well as the number of doctor visits are significantly increased in AD caregivers (Kiecolt-Glaser et al., 1991). One mechanism for the immune dysfunction in response to chronic stress is a reduction of telomeres and telomerase activity, potentially leading to early immune senescence (Epel et al., 2004). Mice with naturally elevated anxiety levels have increased activated microglia and perivascular macrophages in the brain, than less anxious strains (Li et al., 2014), suggesting that anxiety can increase the inflammatory background in the brain.

The acute effects of systemic inflammation upon cognition and behavior are not limited to the elderly or the critically ill. As we have witnessed in ourselves and those near us, even a minor and self-limited common cold induces a transient syndrome known as *sickness behavior* (SB) marked by fatigue, depression, lack of drive, malaise, sleep disturbances, decreased physical activity, and social interactions, as well as cognitive impairment (Capuron et al., 1999, 2001). Healthy volunteers develop anxiety, depression, and memory impairment in response to a low dose of lipopolysaccharide (LPS), and the development of such clinical scenario correlates with TNF secretion (Reichenberg et al., 2001). Some chronic infections may go unrecognized for long periods, as is the case in tuberculosis, human immunodeficiency virus (HIV), hepatitis B virus (HVB), or hepatitis C virus (HCV). Unlike septic patients, patients with chronic infections have an organized and targeted immune response (versus a massive and diffuse one in sepsis), even if the response is ineffective to clear the infection. Those patients, however, have increased cognitive and behavioral problems. For instance, patients with HCV infection have increased rates of fatigue and depression, as well as evidence of metabolic brain dysfunction in the absence of acute hepatitis (Forton et al., 2001, 2002; Hilsabeck et al., 2002). Patients with chronic HCV that have been treated with pegylated interferon (IFN)-α develop significantly higher incidence of depression when compared to the patients’ state before IFN-α administration (Reichenberg et al., 2005). This supports the role of large loads of inflammatory cytokines in inducing and sustaining brain dysfunction. Experimentally, NADPH oxidative activity and nitric oxide synthase (iNOS) are induced in the brain shortly after systemic inflammation (Wong et al., 1996; Yokoo et al., 2012), potentially leading to NMDA-dependent neurotoxicity (Dawson et al., 1993). Evidence derived from post-mortem studies indicates that iNOS is also induced in the brain of patients dying of severe sepsis, but the role of iNOS as inductor of neurotoxicity in response to systemic inflammation has not been assessed in patients surviving severe sepsis (Sharshar et al., 2003). Experimentally, preemptive administration of the free radical scavenger endarvone before sepsis induction resulted in reduced neuronal damage and blood–brain barrier (BBB) permeability (Yokoo et al., 2012). Administration of the antioxidants *N*-acetylcysteine and deferoxamine shortly after murine sepsis induction has shown long-term neuroprotective effects (Barichello et al., 2007).

## Immunity in Sepsis

The immune system is in a constant state of surveillance against potential pathogens and self-generated molecules indicative of damage. Under normal conditions, inflammation is a well-orchestrated response with constant fine-tuning. Once microorganisms have breached the skin and mucosal barriers, innate immunity is critical in preventing further invasion by launching inflammation. After the infection source has been cleared, the inflammatory response also plays an important role in tissue repair and functional healing. When the source of damage has been controlled, the same mechanisms that initiated and regulated inflammation will dampen the response. Large loads of pathogens, or infection by highly virulent pathogens, can trigger an *en-masse* systemic response that leads to sepsis and multiple organ failure (Deutschman and Tracey, 2014). Moreover, during sepsis the inflammatory response does not resolve for several days or weeks (Valdés-Ferrer, 2014), leading to persistent release of high-mobility group box-1 (HMGB1) (Valdés-Ferrer et al., 2013b). HMGB1 is a highly conserved nuclear protein that can be passively released by stressed cells, or actively secreted by monocytes and other immune cells in response to inflammatory signals, playing a critical role in the innate immune response to inflammatory and sterile injury alike (Andersson and Tracey, 2011). HMGB1 in turn primes resident monocytes toward an inflammatory, cytokine producing phenotype (Valdés-Ferrer et al., 2013a). Severe trauma, as well as surgery can lead to large loads of endogenous pro-inflammatory molecules (damage-associated molecular patterns (DAMPs) being released. A few DAMPs have been shown to induce brain dysfunction *in vivo*. Of those, TNF and IL-1 can mediate long-standing cognitive and behavioral changes and, in experimental settings, interfering with the effect of TNF reduces the effect of trauma in the formation of contextual memory (Terrando et al., 2010b). A number of genes regulating immune responses are also closely related to neurodegenerative diseases (Table 1).

**Table 1:** **Major immune genes associated with neurodegenerative diseases**.

Gene/protein	Disease (reference)	Function	Expressing cells	Consequence of mutation
TREM2	AD (Guerreiro et al., 2013; Jonsson et al., 2013), PD (Rayaprolu et al., 2013), FTD (Rayaprolu et al., 2013), Nasu–Hakola disease (Paloneva et al., 2002)	Anti-inflammatory, pro-phagocytic	Myeloid cells	Loss-of-function (Kleinberger et al., 2014)
TYROBP	AD (Zhang et al., 2013), Nasu–Hakola disease (Paloneva et al., 2000)	Binding partner of TREM2	Immune cells	Loss-of-function
CD33	AD (Paloneva et al., 2002; Bertram et al., 2008)	Anti-inflammatory, anti-phagocytic (Bradshaw et al., 2013; Griciuc et al., 2013)	Myeloid cells	Increased expression and activation (Bradshaw et al., 2013)
TREX 1	NPSLE (de Vries et al., 2010), SLE (Lee-Kirsch et al., 2007b), AGS (Crow et al., 2006), familial chilblain lupus (Lee-Kirsch et al., 2007a)	Cytosolic DNA clearance (Stetson et al., 2008)	Ubiquitous	Loss-of-function
CSF1R	HDLS (Rademakers et al., 2012)	Microglial proliferation and differentiation (Stanley et al., 1997)	Myeloid cells	Loss-of-function
SOD1	fALS (Rosen, 1993)	O^2−^ scavenging	Ubiquitous	Loss-of-function (Ghadge et al., 1997; Pasinelli et al., 1998)
GRN	Tau negative FTD (Baker et al., 2006)	Secreted chemoattractant factor, pro-phagocytic (Pickford et al., 2011), anti-inflammatory inflammation (Martens et al., 2012)	Widely expressed	Loss-of-function (Chen-Plotkin et al., 2010)
CR1	AD (Lambert et al., 2009)	Pro-phagocytic	Widely expressed	Unknown, loss-of-function? (Wyss-Coray et al., 2002)
HLA–DRB5	PD (International Parkinson Disease Genomics Consortium et al., 2011), AD (Yu et al., 2015)	Antigen presentation	Antigen presenting cells, inducible on other cell types (Ting and Trowsdale, 2002)	Unknown, deregulation of inflammatory response?

*AD, Alzheimer’s disease; AGS, Aicardi–Goutières syndrome; CR1, complement receptor 1; fALS, familial amyotropic lateral sclerosis; FTD, frontotemporal dementia; GRN, granulin gene; PD, Parkinson disease; NPSLE, neuropsychiatric SLE; HDLS, hereditary diffuse leukoencephalopathy with spheroids; SOD, super oxide dismutase; TREM, triggering receptor expressed on myeloid cells; TYROBP, TYRO protein tyrosine kinase-binding protein*.

## Cerebral Consequences of Systemic Inflammation: What We have Learned from Sepsis and Other Inflammatory Conditions

**Figure 1:**
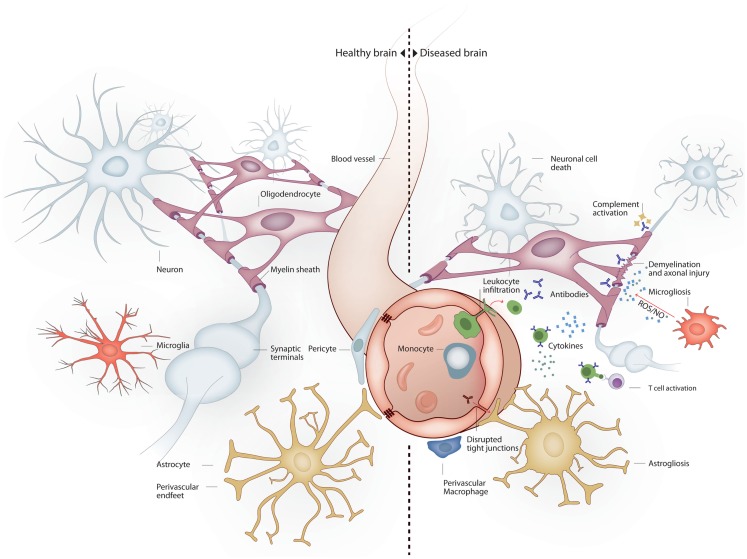
**Brain milieu changes in response to systemic inflammation**. Under healthy conditions the main cell types present in the brain are neurons, oligodendrocytes, astrocytes, and microglia. Neurons connect to each other through long axonal processes with synapses. Oligodendrocytes support axons with myelin sheaths. Astrocytes interact with blood vessels to form the blood–brain barrier and maintain neuronal synapses. Microglia form long processes that surveillance the brain and phagocytose apoptotic cells and prune inactive synapses without induction of inflammation. Under inflammatory conditions several mechanisms lead to neurodegeneration. Peripheral immune cells and inflammatory molecules traverse the blood–brain barrier exerting direct and indirect neuronal cytoxicity. Oligodendroglial myelin sheaths can be affected leading to axonal degeneration. Astrocytosis leads to reduced blood–brain barrier and synaptic maintenance. Microgliosis leads to a pro-inflammatory microglial phenotype with reduced phagocytic and tissue maintenance functions.

**Figure 2:**
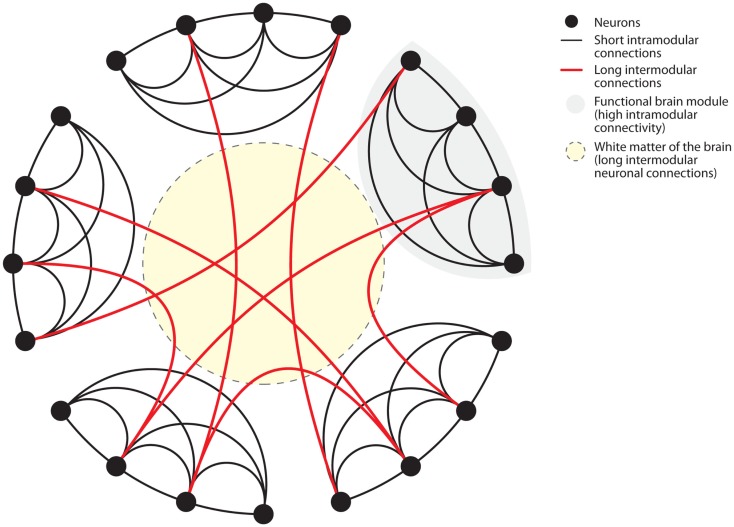
**Connectome of the human brain**. The human brain is organized as a small-world network. Neurons (black dots) form functional modules (gray shaded area). Within such modules, high connectivity is established by short intramodular connections (black lines). Additionally, long intermodular connections located in the white matter (red lines in yellow shaded area) connect different modules with each other. Small-world networks ensure parallel processing of different modes of information within specialized functional modules. Long intermodular connections (red lines) integrate different kinds of information to code a complex response by the brain. The “wiring cost” of neuronal connections is determined by the energetic requirements to maintain these connections. Short intramodular connections have low wiring costs (black line). Long intermodular connections ensure high network efficiency through parallel information processing at the expense of high wiring costs (red line). High wiring cost renders long intermodular connections (red lines) susceptible to energetic imbalance caused by systemic inflammation. Figure modified after Watts and Strogatz (1998).

The nervous system is particularly vulnerable to damage in response to systemic inflammation. Inflammation-induced infiltration of immune cells and mediators into the brain leads to profound structural and functional changes (Figure 1). As a consequence, up to 81% of septic patients develop sepsis-associated delirium (SAD) (Ely et al., 2001, 2004), with elderly patients being at particularly high risk (McNicoll et al., 2003; Iwashyna et al., 2012). In the elderly, severe sepsis is sufficient to trigger new cognitive decline of sufficient importance as to profoundly interfere with quality of life (Iwashyna et al., 2010). SAD is not only common in septic patients but also is a reliable indicator of bad prognosis (Eidelman et al., 1996). Six-month mortality among septic patients is twice as high in patients who develop SAD at any point in time during hospitalization (Ely et al., 2004). Altered mental status is more common in septic patients with bacteremia than in those with negative blood cultures (Eidelman et al., 1996). Magnetic resonance imaging (MRI) in patients with septic shock has shown that new white matter lesions are common (Sharshar et al., 2007). Neonatal sepsis is also marked by abnormalities of the white matter (66% of infants in one cohort), and white matter lesions correlate to poorer mental and psychomotor development at 2 years (Shah et al., 2008). The burden of white matter damage worsens with duration of shock, indicating that sepsis interferes with brain connectivity (Figure 2). Gray matter damage in response to sepsis is far less clear. Imaging and post-mortem studies from patients, as well as data derived from experimental models, indicate that gray matter damage is the norm. In comparison to subjects dying of other causes, brain histology from severe sepsis patients shows a higher number of CD68^+^ and major histocompatibility complex (MHC)-class II microglia in the cerebral cortex and the white matter. Moreover, in septic brains ameboid-shaped activated microglia can be found in gray and white matter alike (Lemstra et al., 2007). An MRI study of premature infants showed reduced volume of deep gray matter structures, and although the findings were consistent in the small subset of septic infants, those were not different from premature non-septic infants (Boardman et al., 2006).

In experimental sepsis, persistent cognitive impairment has been observed in rats completely recovered and with negative blood cultures. This indicates that clearing the trigger of sepsis does not prevent the appearance of persistent brain damage (Barichello et al., 2005). Brain mitochondria become dysfunctional during experimental sepsis, showing increased proton permeability, inadequate membrane potential recovery, and reduced oxidative phosphorylation (d’Avila et al., 2008). In mice, the BBB becomes leaky within 24 h following sepsis induction (Yokoo et al., 2012). While the number of hippocampal neurons is not reduced in mice surviving abdominal sepsis, there is neuronal degeneration (Yokoo et al., 2012), and the spine density of CA1 neurons is significantly diminished, and this finding correlates with circulating HMGB1 (Chavan et al., 2012). Anti-HMGB1 neutralizing monoclonal antibodies improve the memory deficit observed in experimental sepsis, although the mechanism of neuroprotection behind this protective effect is still under investigation. In response to systemic LPS, cortical mRNA expression of IL-1β, IL-6, TLR2, TLR4, Scavenger A, and glial fibrillary acidic protein (GFAP) are upregulated within 4 h, indicating that cortical inflammation and glial activation occur in parallel or shortly after systemic inflammation ensues (Noh et al., 2014; Silverman et al., 2014). LPS also increased brain mitochondrial complex II/III activity, and reduced brain glutathion levels within 1 h after systemic administration, supporting the role of systemic inflammation in cerebral mitochondrial dysfunction in sepsis (Noh et al., 2014). Interestingly, even a single and relatively low dose of LPS (that is sufficient to cause around 10% mortality) has been shown to have long-term behavioral and cognitive consequences. In one study, 30-day evaluation showed increased anhedonia and anxiety, altered working memory, as well as reduced exploratory behavior (Anderson et al., 2015). Consistent with that, in a model of endotoxemia in aged rats, a single systemic injection of LPS induced brain inflammation that lasted for at least 30 days. Interestingly, those rats had only transient increase of circulating TNF and IL-1 lasting <6 h in response to the injection (Fu et al., 2014). The hippocampus and dentate gyrus were particularly affected, showing astrogliosis and increased TNF, IL-1, and NF-κB mRNA and protein levels. This suggests that even transient bouts of systemic inflammation of only limited significance can cause sustained brain damage.

Delirium and brain dysfunction can be induced directly by soluble mediators such as TNF, IL-1, or HMGB1, as well as indirectly through activation of microglia and astrocytes. LPS has been shown to induce microglial activation and memory impairment in young mice through a mechanism that is at least partially dependent on interleukin (IL)-1, and HMGB1, both occurring within 24 h of LPS administration (Terrando et al., 2010a). However, while the rapid effect of LPS upon brain homeostasis is clear, a single sublethal injection of LPS (5 mg/kg) is sufficient to impair behavior and memory, mediated by a reduction in neural stem cell proliferation in the dentate gyrus, as well as inducing microglia invasion and activation to the hippocampus, all lasting for at least 30 days (Anderson et al., 2015). Minocycline, a tetracycline derivative used commonly for acne and other infections, inhibits activation and proliferation of microglia (Tikka et al., 2001). Intracerebroventricular (ICV) administration of minocycline immediately after sepsis induction by CLP reduced cerebral inflammation, decreased BBB permeability, and protected against memory impairment observed in untreated septic mice (Michels et al., 2015).

## Susceptible Brain: Predisposing Factors Drive Neurodegeneration

While the nervous system is susceptible to peripheral challenges, one question under active research concerns to the role of a *susceptible* nervous system. Genes and molecular factors rendering rodents and humans prone to neurodegeneration have been identified recently. Genes predisposing to neurodegeneration are summarized in Table 1. The mouse strain DBA/2J has a natural anxious behavior. In response to a systemic challenge with low-dose LPS (1 mg/kg), DBA/2J mice develop increased anxiety, and increased expression of hypothalamic mRNA expression of the inflammatory genes *Il1b*, *Il6*, *tnf*, and *Nos2* in comparison to behaviorally normal strains (Li et al., 2014), suggesting that emotional stress has a role in magnifying a systemic inflammatory stimulus.

Sepsis-induced cognitive decline can be exacerbated in individuals with a susceptible brain (Iwashyna et al., 2010). Interestingly, in community-based patients with AD, even mild inflammatory conditions can lead to cognitive decline and disease progression (Holmes et al., 2009). Similarly, in a mouse model of AD, within 24 h after the systemic administration of LPS, cerebral IL-1β and IL-6 were induced, followed by changes in β-amyloid precursor peptide (APP) isoforms similar to those observed in AD patients (Brugg et al., 1995). In the past few years, possible mechanisms of damage have been proposed based on rodent models. In contrast to healthy control mice, scrapie-infected mice – a model of prion disease – show markedly enhanced susceptibility to LPS, leading to altered working memory, synaptic loss, enhanced expression of inflammatory receptors, and microglial activation (Murray et al., 2012). Cognitive and motor coordination are more severely impaired in mice that have been scrapie-infected for longer time periods, and one-time inflammation seems to accelerate disease features of neurodegeneration (Cunningham et al., 2009). Moreover, in prion disease neurodegeneration occurring in response to systemic inflammation is proportional to the burden of preexisting neurodegeneration. In a rat model of toxin-induced Parkinson disease (PD) induced by 6-hydroxydopamine, persistent systemic inflammation induced by IL-1β resulted in a reduction in the number of neurons, as well as an increase in activated microglia in the substantia nigra (SN) (Pott Godoy et al., 2008). The damage to SN in response to inflammation is not limited to susceptible animals. In response to systemic LPS, 8-month-old wild type mice showed a rapid reduction of tyrosine hydroxylase (dopaminergic) neurons, with a reciprocal expansion in activated microglia in the SN occurring shortly thereafter (Reinert et al., 2014). Amyotrophic lateral sclerosis (ALS) is a neurodegenerative disorder characterized by progressive, irreversible, and highly lethal motor neuron damage. In transgenic mice expressing a mutant form of superoxide dismutase 1 (SOD1), an experimental model of ALS, inflammation in the anterior horns of the spinal cord is a hallmark. Inflammation is driven by tissue-resident microglia displaying a neurodegeneration-specific pattern of gradual increase in insulin-like growth factor 1 (*Igf1*) and the tyrosine kinase receptor AXL (*Axl)* transcription that closely correlate with disease progression (Chiu et al., 2013b). The activated microglia induce motor neuron death through induction of NF-κB (Frakes et al., 2014). The peripheral nervous system of SOD1 mutant mice also displays inflammation that is proportional to disease progression; in this case, there is invasion of the anterior (motor) roots by circulation-derived active macrophages (Chiu et al., 2009). While spinal cord inflammation may be common in SOD1 mutant mice, those mice display an increased susceptibility to chronic systemic inflammation, leading to disease progression, induction of TLR2, early axonal damage, and accelerated mortality (Nguyen et al., 2004). Although the mechanism for increased susceptibility to systemic inflammation in SOD1 mutant mice is unknown, it is possible that persistent systemic inflammation can further activate microglia and induce the translocation of NF-κB and activation of downstream inflammatory mediators.

Altogether, current evidence indicates that cognitive impairment is common in sepsis; that a susceptible brain is fertile ground for systemic inflammatory-induced dysfunction; and that cognition and behavior are persistently challenged, even after resolution of acute sepsis.

## Systemic Lupus Erythematosus: Systemic Autoimmune Inflammation as Driver of Neurodegeneration

Systemic lupus erythematosus (SLE) is a chronic relapsing–remitting autoimmune disease that affects multiple organs and often involves the central nervous system (CNS). It has a high female preponderance and occurs mainly in women of childbearing age. Immunologically, it is characterized by a loss of tolerance to self-antigens and abnormal B- and T-cell responses. Immunoglobulin complexes can deposit in tissue and can cause systemic inflammation. Anti-nuclear antibodies can be found in up to 98% of patients, yet can also be detected in other autoimmune conditions. Neuropsychiatric SLE (NPSLE) is an incompletely understood medical problem, and its clinical picture diverse. Depending on the study, the percentage of patients with neurological and psychiatric involvement can vary from 12 to 95% (Ainiala et al., 2001). CNS involvement indicates a more severe clinical presentation of SLE (Mak et al., 2012). Due to the wide range of motor, sensory, cognitive, and behavioral symptoms, diagnosis of neuropsychiatric symptoms is challenging (Jeltsch-David and Muller, 2014). Memory impairment is linked to neuronal cell death in the hippocampus, and might be caused by antibody mediated cytotoxicity following binding to neuronal cell surface receptors, such as the NMDA receptor (Faust et al., 2010) or neuronal surface P antigen (NSPA) (Bravo-Zehnder et al., 2015). Cognitive impairment in NPSLE has been associated with a higher frequency of hippocampal atrophy (Appenzeller et al., 2006). There is a great set of data demonstrating that neuropsychiatric symptoms could be caused by autoantibodies, cytokines or microvasculopathy, and thrombosis. The most common brain abnormalities in patients include microvasculopathy, which can be caused by antiphospholipid antibodies that bind to clotting factors and endothelial cells (Belmont et al., 1996). In addition to vasculopathy, post-mortem brain studies of patients show infarcts and hemorrhages, cortical atrophy, ischemic demyelination, as well as CNS demyelination (Hanly et al., 1992). Currently, it is not known whether CNS involvement develops independently or occurs as a consequence of systemic organ dysfunction or both. Neurological involvement might be related to treatment, infection, or metabolic disorders or may be part of a coexisting disease. Increased BBB permeability leads to CNS penetration of immunoglobulin, pro-inflammatory cytokines, and albumin. A limited number of studies addressed the genetics of NPSLE, and found an increased susceptibility in patients with brain involvement compared to SLE sparing CNS involvement. Particularly TREX 1 (DNase III) gene mutations have been reported in NPSLE (Table 1; Lee-Kirsch et al., 2007b; de Vries et al., 2010), a mutation that is also found in other brain diseases and is involved in apoptosis and oxidative stress. TREX 1 knockout mice develop severe inflammatory myocarditis, resulting in reduced survival rates (Morita et al., 2004) due to accumulation of single stranded DNA fragments, which facilitates the production of type 1 IFN (Miner and Diamond, 2014). Larger genome wide association studies (GWAS) comparing patients with and without neuropsychiatric involvement are needed to further understand NPSLE. Despite improved imaging and the availability of potential biomarkers (autoantibodies, cytokines, chemokines), NPSLE remains a diagnostic dilemma. So far, no specific treatment is available for NPSLE; however, the CD20 B-cell targeted treatment rituximab was shown to be promising in a small cohort of refractory NPSLE patients (Tokunaga et al., 2007). In addition, peptide mimotopes in patients with anti-brain reactive antibody responses may hold promise (Bloom et al., 2011).

## Multiple Sclerosis: Brain Inflammation as Driver of Neurodegeneration

Multiple sclerosis (MS) is the most common inflammatory demyelinating diseases in young adults with a high risk of long-term disability affecting over 2.5 million people worldwide (Compston and Coles, 2008). Despite advances in diagnosis and treatment, the cause of MS remains unknown. While effective treatment for the relapsing–remitting form has improved significantly, treatment for the progressive disease course is still of limited utility (Lassmann et al., 2007). MS is presumably caused by an exogenous trigger in genetically predisposed individuals (Sospedra and Martin, 2005b), resulting in inflammation, demyelination, and neurodegeneration. MS is initiated by autoreactive T-cells against a yet unknown CNS antigen (Sospedra and Martin, 2005a). In addition to T-cells, in the last years, an important role of B-cells and antibodies has re-emerged as major contributors to the disease (Hauser et al., 2008). Experimental autoimmune encephalitis (EAE), the most widely used animal model for studying MS (Friese et al., 2006) is either induced by injection of myelin derived proteins together with adjuvant or by adoptive transfer of CD4^+^ encephalitogenic T-cells. It has been increasingly recognized that this model does not accurately represent the full spectrum of the human disease. MS is a heterogeneous disease, with patients experiencing a broad range of motor, cognitive, and neuropsychiatric impairment (Chiaravalloti and DeLuca, 2008). Approximately 80% of patients develop relapsing–remitting MS (RRMS), where patients experience relapses that are followed by partial or complete remission (Sospedra and Martin, 2005a). In an advanced disease stage, the majority of RRMS patients convert to secondary progressive MS (SPMS), with a steady disease progression in the absence of relapses and remissions (Sospedra and Martin, 2005b). A minority of patients (15%) suffer from an early onset progressive neurological decline, defined as primary progressive MS (PPMS) (Sospedra and Martin, 2005a,b; Miller and Leary, 2007). PPMS presentation is similar to that of SPMS patients (Confavreux and Vukusic, 2006). Since the progressive disease is not associated with the number of relapses or the extend of inflammation (Friese et al., 2014), it remains unknown what drives neurodegeneration in MS.

So far, the interplay, timing, and localization of inflammation, breakdown of the BBB, demyelination, axonal dysfunction, neurodegeneration, gliosis, atrophy, and repair mechanisms are incompletely understood (Figure 1) (Noseworthy et al., 2000; Bruck, 2005). The pathological hallmark of MS is CNS *plaques*, which are areas of focal demyelination in the white matter (Popescu and Lucchinetti, 2012). Whereas glial scarring was described as a characteristic feature of demyelinating plaques, most studies used to emphasize an initial preservation of axons. During RRMS, focal lesions, which are disseminated in space and time (McFarland and Martin, 2007), are associated with a BBB breakdown and an infiltration and activation of immune cells (Sospedra and Martin, 2005b). Lesions can be completely or partly resolved due to remyelination and resolution of inflammation. In contrast, the progressive disease stage results in irreversible deficits and is pathologically characterized by chronic axonal degeneration and gliosis. Although demyelination is described as primary event in MS, and neurodegeneration is believed to occur as a secondary event, it is now widely accepted that axonal loss occurs already in acute white matter plaques (Bruck and Stadelmann, 2003). MS was believed to be primarily a white matter disease, yet cortical gray matter lesions and atrophy in the brain and spinal cord can be observed at different time points of the disease, even as early as at the time of diagnosis (Peterson et al., 2001; Bø et al., 2003; Kutzelnigg et al., 2005, 2007; Geurts et al., 2007; Barkhof et al., 2009). In addition, the marker for neuronal integrity *N*-actetylaspartate (NAA) is decreased throughout the CNS at an early disease stage (De Stefano et al., 2001). NAA levels are also decreased in the normal appearing white matter and in the cortical gray matter. Increased concentrations of the neurofilament protein in the cerebrospinal fluid (CSF) can be observed at all stages of the disease, which additionally reflects early ongoing neurodegeneration (Kuhle et al., 2011).

While there has been major progress in treating RRMS with anti-inflammatory and immunomodulatory treatment, there are currently no effective treatment options for the progressive disease. The inefficiency of anti-inflammatory treatment in PPMS and SPMS was explained by the lack of inflammation during neurodegeneration. However, this could be due to impaired drug delivery to the brain due to a widely restored BBB integrity in the progressive disease stage (Lassmann et al., 2012). In contrast to the traditional view that neurodegeneration in MS occurs in the presence of limited or almost absent inflammation, it has been shown that inflammation is present at all stages of the disease (Frischer et al., 2009). In fact, there is a massive infiltration of microglia and immune cells into gray matter lesions, including the deep gray matter (Lucchinetti et al., 2011). However, chronic lesions in the gray matter show less extend of immune cell infiltration compared to chronic white matter lesions (Popescu and Lucchinetti, 2012). Cortical lesions can be observed even before detection of white matter lesions. Most patients have the highest prevalence of cortical lesions in the progressive disease stage, where diffuse meningeal inflammation correlates with the disease severity, suggesting that cytokines, chemokines, reactive oxygen species (ROS), and glutamate released by meningeal infiltrating immune cells contribute to neurodegeneration by disturbing the neuronal metabolic pathway (Friese et al., 2014). A redistribution of ion channels could be a compensatory effect of the inflamed axon but finally accelerates neurodegeneration.

Overall, the cause and mechanism of neurodegeneration in MS is unresolved, yet it is highly likely that there is a large variability within individual subgroups of MS patients. In some patients, inflammation can be caused by primary neurodegeneration, whereas demyelination can be the primary cause in others, causing axonal transection and thereby initiating Wallerian and retrograde neurodegeneration (Friese et al., 2014). It has been shown that demyelination can lead to axonal degeneration and neuronal loss by limited trophic support of oligodendrocytes (Fünfschilling et al., 2012; Lee et al., 2012). Neurodegeneration in MS can also occur, at least partly, in the absence of demyelination since axonal and neuronal injury in the normal appearing white matter is rather associated with global CNS inflammation but not with white matter demyelination (Friese et al., 2014). The continuous worsening of the disease can be explained by progressive neuronal loss, which cannot be compensated over time by protective repair mechanisms. However, the total lesion load does not correlate with the extent of neurodegeneration, suggesting that neurodegeneration is the result of inflammatory processes in non-lesioned white matter. Alternatively, MS can be a primary neurodegenerative disease rather than an autoimmune disease (Trapp and Nave, 2008). However, genetic findings show no correlation of genetic risk alleles in MS with other neurodegenerative diseases. A role of genetic heritability in MS was supported by early findings, which observed aggregations of MS cases in some families as well as an increased prevalence of MS in monozygotic twins compared to dizygotic twins (Ebers et al., 1995). Genetic susceptibility genes have been described but the disease does not follow a Mendelian inheritance pattern but a rather complex pattern of interaction. The most striking findings regarding genetic susceptibility in MS comes from studies obtained in the 1970s showing an association of MS with alleles of the MHC, particularly HLA-DRB1*15, of the class II gene HLA-DRB1, which is the most important risk allele in MS. GWAS and meta analysis enabled the discovery of many single nucleotide polymorphisms (SNIP) in MS. To date, around 350 MHC and non-MHC loci have been identified (Wang et al., 2011), which are mainly involved in immunological processes (Wang et al., 2011). Some of those risk genes overlap with other autoimmune diseases, whereas other risk genes are unique to MS. Based on the current studies, the genetic risk for MS is not linked to a single gene mutation but rather caused by a complex interplay of many SNIP, which amplify and result in small or moderate risk effects. MS is probably caused by a multifactorial pattern of inheritance, which needs further investigation in individual larger patients groups. Once GWAS studies identify SNIPs in MS patients, there is a great interest to correlate these findings with functional data. Prime examples are the findings of SNIPS in the *TNFRSF1A* gene that have been associated with a worse clinical outcome in MS patients. This mutation results in a novel soluble splice form of the TNF receptor (TNFR1) that, in contrast to the membrane bound form, lacks NF-κB activity and apoptotic activity but can block the function of TNF and thus mimics anti TNF therapies, which exacerbate MS (Gregory et al., 2012).

So far, most genetic studies focus on genes responsible for the initiation of the disease rather than on genes influencing the severity of the disease (Friese et al., 2014). Thus, future studies are needed to identify genes that trigger the progression of the disease in the presence of inflammation. One study showed that meningeal inflammation is associated with small fiber axonal loss in the spinal cord of patients that were HLA-DRB1*15 positive, correlating neurodegeneration with increased genetic susceptibility (DeLuca et al., 2013). Another study demonstrated higher glutamate levels in the brain of MS patients harboring certain risk alleles for genes involved in the glutamate pathway (Baranzini et al., 2010). A polymorphism of the inositol polyphosphate-4-phosphatase, type II (Inpp4b), which was described for MS results in decreased nerve conduction velocity, which could aggravate the disease (Lemcke et al., 2014). More GWAS studies are needed to confirm these results and there should be a particular focus on polymorphisms associated with the progressive disease stage. In addition to genetic susceptibility genes, several environmental factors have been suggested to trigger the disease, such as vitamin D, smoking, Epstein–Barr virus infection, and geographical location in relation to latitude gradient (Ascherio and Marrie, 2012).

## Mechanisms of Neurodegeneration: Systemic Inflammation Drives Disruption of Brain Networks

Biological systems, such as the neuronal network of the human brain (Sporns et al., 2005; Achard et al., 2006; Bullmore and Sporns, 2009) have “small-world” properties (Watts and Strogatz, 1998). Small-world networks have two levels of organization (Figure 2). On the local level, groups of neurons specialized in a specific task form functional modules with high short intramodular connectivity. On the global level, different modules are connected through long intermodular connections. The advantage of the latter type of connections is enhanced computational efficiency through parallel processing of information (Watts and Strogatz, 1998; Latora and Marchiori, 2001; Bullmore and Sporns, 2009). Anatomically, long intermodular connections are formed by axonal fiber tracts in the white matter (Bullmore and Sporns, 2009; Toga et al., 2012). Long fibers are characterized by high energetic “wiring costs” (Bullmore and Sporns, 2012). To provide the energy for the maintenance of these long fibers the brain is relying on a constant energy supply. Recent findings have elegantly identified oligodendrocyte-derived lactate as the main energetic substrates for axonal maintenance (Fünfschilling et al., 2012). Consistently, disruption of this oligodendrocyte-neuronal metabolic coupling triggered neurodegeneration (Lee et al., 2012). Systemic inflammation poses dramatic challenges to the energetic supply of the brain.

To cover its *wiring costs* the brain is highly reliant on a constant nutrient supply. Nutrient supply through blood vessels can be compromised through vascular pathologies associated with systemic inflammation. During severe sepsis, disseminated intravascular coagulation leads to diffuse intravascular formation of thrombi and hemorrhages due to depletion of coagulation factors. The consequences are diffuse ischemic foci throughout the body and dysfunction of affected organs. Infarctions or hemorrhages occurring in the course of long connecting tracts in the brain lead to disconnection of distant brain regions and reduced efficiency of neuronal networks. These changes might contribute to white matter lesions observed in MRI studies of acute sepsis cases (Sharshar et al., 2007) and sepsis survivors (Morandi et al., 2012; Semmler et al., 2013). Mitochondrial dysfunction is another complication of sepsis affecting brain function. Despite normal or even increased tissue oxygen availability (Boekstegers et al., 1991), oxygen utilization is drastically reduced in critically ill patients leading to multi-organ failure and mortality (Brealey et al., 2002). Brain mitochondrial dysfunction was shown in rodent models of sepsis (d’Avila et al., 2008). This hibernation-like metabolic state was proposed as a physiological protective tissue reaction (Singer et al., 2004). However, mitochondrial dysfunction might persist in sepsis survivors (Comim et al., 2011). The cause for mitochondrial dysfunction might be a combination between induction of ROS during sepsis (Wong et al., 1996) and impaired mitochondrial turnover (Singer, 2014). Taken together, the complicated interrelation between focal hypoperfusion and reduced oxygen utilization lead to a complex acute and chronic phenotype referred to as sepsis-associated encephalopathy (Gofton and Young, 2012; Sonneville et al., 2013).

Autoimmune disorders have a chronic course of vascular pathology with acute flares. The most common vascular pathology is the autoantibody-associated antiphospholipid syndrome (Ben Salem, 2013; Giannakopoulos and Krilis, 2013; Million and Raoult, 2013). Patients with antiphospholipid syndrome display cognitive deficits (Gómez-Puerta et al., 2005; Tektonidou et al., 2006). MRI studies found diffuse infarctions and white matter lesions in these patients (Gómez-Puerta et al., 2005; Tektonidou et al., 2006; Valdés-Ferrer et al., 2008). The role of mitochondrial dysfunction in autoimmune diseases is not clear. In SLE, mitochondrial dysfunction and ATP depletion were shown as triggers of lymphocytic cell death (Gergely et al., 2002). An interesting view of mitochondrial dysfunction in the context of MS was recently proposed (Lassmann, 2011). In line with the concept of high “wiring costs” imposed on the brain by long intermodular connections (Bullmore and Sporns, 2012), Hans Lassmann argues that inflammation in MS causes mitochondrial damage and inability of the brain to maintain neuronal processes. The source of mitochondrial damage is radicals formed as a consequence of inflammation in MS (Lassmann, 2011; Fischer et al., 2012). Disruption of neuron–glia metabolic coupling might be another potential mechanism causing neurodegeneration and network disruption (Allaman et al., 2011; Lee et al., 2012).

Taken together these findings indicate that systemic inflammation leads to an energy crisis of the brain that reduces its connectivity. Oxidative stress might be the main mediator of this pathology. Thus, inflammation-induced changes in the brain resemble hallmarks of the aged brain where oxidative damage leads to decreased expression of genes associated with synaptic plasticity and increased expression of stress-response genes (Lu et al., 2004). Likewise, the brain during systemic inflammation shows hallmarks of neurodegenerative diseases where oxidative stress and mitochondrial damage have consistently been found (Lin and Beal, 2006). Studies with bigger sample sizes are needed to identify common mechanisms between systemic inflammation, brain aging, and neurodegeneration.

## Systemic Inflammation-Associated Immunopathology Irreparably Damages the Architecture of the Brain

With approximately 90 billion neuronal and non-neuronal cells, respectively (Azevedo et al., 2009), the human brain is characterized by high architectural complexity (Braitenberg and Schüz, 1991). Due to this complexity, the repair capacity is limited rendering the human brain highly susceptible to tissue damage. As primary preventive measures the brain is protected from different modes of tissue damage; for example, by the skull bone from mechanical damage or by the BBB from blood-borne pathogens. The BBB is mainly formed by endothelial cells and astrocytes (Abbott et al., 2006; Weiss et al., 2009); the formation of tight junctions between endothelial cells forms a highly selective barrier that becomes more permeable during systemic inflammation (McColl et al., 2008; Weiss et al., 2009). A third kind potential source of brain tissue damage is the immune system itself. As pathogen defense is invariably associated with host tissue damage (Graham et al., 2005) an anti-inflammatory milieu preserves the brain from aberrant immune activation. Under physiological conditions, astrocytes and neurons actively modulate the activation of brain immune cells (Neumann, 2001; Tian et al., 2012). Through this cross-talk brain cells can actively recruit immune cells for purposes of brain homeostasis such as synaptic plasticity, induction of inflammation, clearance of debris, and resolution of inflammation.

Brain-resident microglia and peripheral immune cells maintain immune surveillance of brain parenchyma, CSF, and perivascular space for infectious agents or damage-associated milieu changes (Ousman and Kubes, 2012; Ransohoff and Engelhardt, 2012). In the case of brain infection, complete eradication of some invading pathogens can only be achieved at the cost of irreparable damage to brain tissue. To prevent such damage, the immune system has established active mechanisms of pathogen tolerance (Medzhitov et al., 2012). Examples for coexistence-prone pathogens are herpes simplex virus type I (Khanna et al., 2004) or *Cryptococcus gattii* (Cheng et al., 2009). A growing body of evidence indicates that not only immune tolerance but also resolution of neuroinflammation is a tightly regulated active immunological process (Schwartz and Baruch, 2014). Taken together, anti-inflammatory brain milieu, pathogen tolerance, and resolution of neuroinflammation require a balanced action between different branches of the immune system. An imbalance within the immune system leading to systemic inflammation may be a driver of neurodegeneration through the mechanisms discussed below.

## Caspases as Mediators of Inflammation and Neurodegeneration

Apoptosis is one of the main drivers of neurodegeneration. Apoptosis and cell death constantly occur under physiological conditions throughout the human body and cell debris is cleared by immune cells mostly without induction of chronic inflammation (Green et al., 2009). However, during systemic inflammation, apoptosis of stressed cells might further exacerbate the underlying pathology (Zitvogel et al., 2010). Activators of apoptosis lead to direct or indirect activation of caspases. Interestingly, caspases are not only classical executors of apoptosis (Friedlander, 2003) but also inflammatory caspases are crucial for the activation of the innate immune system through the inflammasome (Martinon and Tschopp, 2004). Caspase-1, as the cleaving enzyme for IL-1β and constituent of the inflammasome, is the prototypic representative of the latter class of caspases (Martinon et al., 2002).

Activation of the innate immune system through the inflammasome is a driver of pathology in age-associated and autoimmune neurodegenerative disorders. In AD, the NLRP3 inflammasome was described a sensor of β-amyloid (Heneka et al., 2013). Of interest, deletion of Caspase-1 or NLRP3 rescued the phenotype in APP/PS1 AD transgenic mice (Heneka et al., 2013). Consistently with that the deletion of the inflammasome scavenger receptor CD36 (Sheedy et al., 2013) ameliorated pathology in the Tg2576 AD mouse model (Park et al., 2013). MS-like lesions were found in humans with mutations of proteins associated with the inflammasome (Compeyrot-Lacassagne et al., 2009). Additionally, NLRP3 and IL-1 knockout mice had decreased pathology following EAE (Matsuki et al., 2006; Gris et al., 2010). Approved clinical treatment options of MS such as IFN β (Guarda et al., 2011) or glatiramer acetate (Burger et al., 2009) have been shown to decrease IL-1β levels, the main cytokine processed by the inflammasome. Additionally, novel evidence extends the functions of the classical apoptotic caspases linking neuroinflammation to neurodegeneration. Activation of microglial caspase-8, -3, and -7 can drive neurodegeneration (Burguillos et al., 2011). Finally, recent evidence from *C. elegans* has described protective effects through mediators of the intrinsic apoptosis pathway against ROS (Yee et al., 2014). Taken together, these finding show an intricate relationship between inflammation and activation of apoptosis. However, the definite role of these mediators in the brain remains to be characterized.

## Microglia- and Macrophage-Derived Microvesicles as Inducers of Neurodegeneration

Cellular components of innate immunity can pack and secrete inflammatory messengers in microvesicles (MVs). Peripheral macrophages, as well as brain microglia can secrete inflammasome components (caspase-1, IL-1β, and IL-18) in MVs, and the presence of extravesicular inflammatory inducers (e.g., astrocitic ATP) is sufficient to induce the neurotoxicity by the inflammatory load of MVs (Bianco et al., 2005; Sarkar et al., 2009; Gulinelli et al., 2012). Although MVs are visible in the CSF in healthy controls, the load of MVs in RRMS, neuromyelitis optica (NMO), brain infections, and brain tumors is significantly increased and, in MS, correlates with disease activity (Verderio et al., 2012). Recent evidence suggest that MVs play a critical role in the spectrum of AD as well. MVs released by activated microglia participate in the neurodegenerative process of AD by promoting the generation of highly neurotoxic soluble forms of β-amyloid (Joshi et al., 2014). Based on this collective evidence, it is now clear that EVs produced by peripheral myeloid cells, as well as immune brain cells, are novel and potentially critical biomarkers for neuroinflammatory conditions by providing a link between inflammation and neurodegeneration.

## Immune Cells and Immune Mediators as Drivers of Neurodegeneration

**Figure 3:**
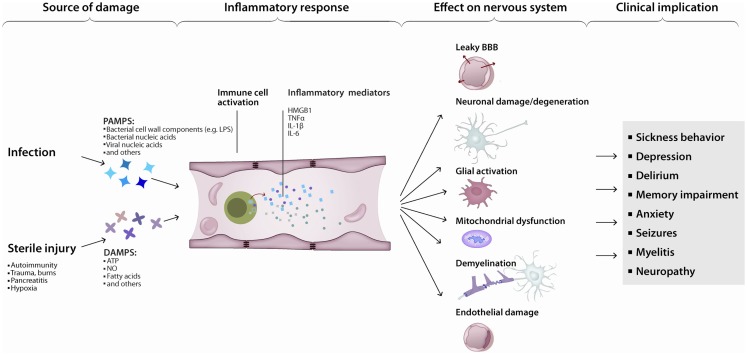
**Inflammation leads to neurodegeneration: a simplified model**. Pathogen- or damage-derived antigens released in sufficient quantity activate systemic inflammation. In turn, peripheral (e.g., monocytes) as well as central (e.g., microglia) immune cells activate, increasing the production and release of inflammatory cytokines, chemokines, and other immunologically active peptides. Those mediators can induce neuronal dysfunction directly or indirectly, by interfering with neuronal homeostasis or disrupting the neuronal milieu. The end-result is a continuum of clinical manifestations from local and transient, to diffuse and persistent.

Various triggers of apoptosis have been described with respect to the brain. Neuronal apoptosis can be directly induced by ROS, pro-inflammatory cytokines or activated immune cells. The consequences of ROS-induced mitochondrial damage on brain metabolism have been discussed above. Additionally, damaged mitochondria are a major source of ROS (Rego and Oliveira, 2003) and mediators of apoptosis (Green and Kroemer, 2004). Conversely, inactivation of ROS has anti-apoptotic effects (Hockenbery et al., 1993; Greenlund et al., 1995). The inflammatory cytokine TNFα (Tamatani et al., 1999; Kaur et al., 2014) and tumor necrosis factor-related apoptosis-inducing ligand (TRAIL) (Aktas et al., 2005) directly induce neuronal apoptosis (Figure 3). Additionally, intracerebroventricularly injected TNFα was shown to induce depression-like symptoms (Kaster et al., 2012). Cytokine mediated induction of apoptosis was also observed by IL-1β (Wang et al., 2005; Kaur et al., 2014). Sources of cytokines under systemic inflammation are brain resident, paravascular or peripheral immune cells (Dantzer et al., 2008). Furthermore, activated immune cells can directly induce neuronal cell death (Figures 1 and 3). Brain-resident microglia convey neuronal toxicity through various mechanisms including secretion of neurotoxic factors (Liu and Hong, 2003; Block et al., 2007), as well as through activation of cyclooxygenase/prostaglandin E2 (COX/PGE2) pathways (Montine et al., 1999; Liang et al., 2005). In fact, blocking the COX/PGE2 pathway by experimentally deleting the prostaglandine receptor EP2 increases mitochondrial degradation of β-amyloid, potentially opening a new therapeutic avenue for AD (Johansson et al., 2015).

Peripheral immune cells can penetrate the BBB under conditions of systemic inflammation (Schmitt et al., 2012) and contribute to brain pathology (Rezai-Zadeh et al., 2009) (Figure 3). Cytotoxic T-cells were shown to be directly neurotoxic in autoimmune and aging-associated neurodegenerative disorders of the CNS (Neumann et al., 2002). Co-localization of T-cells with neurons and neuron-specific cytotoxicity of T-cells was shown *in vivo* and *in vitro* (Giuliani et al., 2003; Nitsch et al., 2004). The pivotal role of pathogenic T-cells in MS is suggested by the fact that adoptive transfer of myelin reactive T-cells is sufficient to induce EAE in rodents. Finally, B-cells have multiple roles in MS (Krumbholz et al., 2012). On the one hand, B-cells can serve as antigen presenting cells for T-cells and B-cell derived cytokines can activate pathogenic T-cells (Schneider et al., 2011). On the other hand, intrathecal IgG synthesis and persistent oligoclonal bands in the CSF are a hallmark finding in MS. Furthermore, antibodies and complement deposition is present in acute MS lesions (Lucchinetti et al., 2000). B-cells were shown to mature in the draining cervical lymph nodes and migrate to the brain (Stern et al., 2014). With advancing pathology, B-cells can be found in serum, brain parenchyma, and meninges of patients (Serafini et al., 2004; Kuerten et al., 2014); and B-cell depletion was shown to be beneficial in a subgroup of MS patients (Hauser et al., 2008).

## Anti-Brain Antibodies as Drivers of Neurodegeneration

B-cell-derived anti-brain antibodies have been identified as drivers of brain pathology in various diseases. In the last decade, an increasing number of anti-brain antibodies has been detected that can affect cognition and behavior (Diamond et al., 2013). For many of these newly discovered antibodies their frequency in disease and involvement in pathogenesis has not yet been determined. Although anti-brain antibodies can be present in around 5% of healthy individuals (Diamond et al., 2013), an intact BBB restricts entry of antibodies into the brain. Under pathological conditions, antibodies may penetrate the BBB through different mechanisms including local and systemic inflammation, or antigen mediated endocytosis (Diamond et al., 2013). In addition, fenestrated endothelial cells of the circumventricular organs, which lack the tight junction of the BBB, may facilitate entry of antibodies into the brain parenchyma (Popescu et al., 2011); or antibodies may be produced intrathecally by B-cells, which migrate into the CNS.

So far, few antibodies have been confirmed to be directly neurotoxic. The most striking evidence is provided by tumor-associated autoantibodies causing paraneoplastic neurological disorders. These antibodies are cross-reactive to neuronal antigens expressed on cancer cells; neuronal pathology is induced through neuronal cell death after antibody binding to neuronal antigens. Clinically, patients present with severe neuropsychiatric symptoms. Rapid removal of paraneoplastic antibodies and surgical removal of the tumor can prevent further neuronal cell death und may reverse neurological pathology. Patients that harbor an antibody response to intracellular antigens have a worse response to treatment compared to patients with antibodies to extracellular neuronal autoantigens. This could be due to an involvement of pathogenic T-cells, which cause irreversible neuronal cell death (Lancaster and Dalmau, 2012).

Anti-NMDA receptor encephalitis is the most common paraneoplastic disorder, with ovarian teratomas as the underlying malignancy in approximately 50% of the patients (Dalmau et al., 2011). These antibodies bind the extracellular domain of the NMDA receptor subunit 1 (GluN1) and cause psychiatric symptoms such as anxiety, memory loss, and psychosis (Dalmau et al., 2007). Binding of antibodies results in reduced levels of NMDA receptor *in vitro* and *in vivo* through antibody mediated capping, crosslinking, and internalization of the receptor (Hughes et al., 2010), which can be reversed upon removal of the antibodies (Seki et al., 2008). Interestingly, patients with high titers of NMDA receptor antibodies have lower levels of the NMDA receptors in postsynaptic dendrites (Dalmau et al., 2008). Direct injection of the antibodies in the cortex of rodents results in excitotoxicity by increased glutamate release (Hughes et al., 2010). A recent study found NMDA receptor antibodies in patients with demyelinating disorders indicating that these antibodies might cause neuropsychiatric symptoms in these patients (Titulaer et al., 2014).

Furthermore, NMDA-receptor-specific antibodies to the subunit 2 (GluN2) have been found in a subset of SLE patients with neuropsychiatric symptoms. These antibodies are cross-reactive to DNA and bind the consensus sequence D/E W D/E Y S/G (DWEYS) present in the extracellular domains of GluN2A and GluN2B subunits of the NMDA receptor (Gaynor et al., 1997). DNA–NMDA receptor antibodies preferentially bind the open configuration of the NMDA receptor and augment NMDA receptor-mediated excitatory postsynaptic potentials (Faust et al., 2010). DNA–NMDA receptor antibodies have been found in serum, CSF, and brain tissue of SLE patients (DeGiorgio et al., 2001; Kowal et al., 2006). Notably, patient derived DNA–NMDA receptor antibodies cause hippocampal neuronal loss and persistent memory impairment in rodents (Kowal et al., 2006). These findings indicate that DNA–NMDA receptor antibodies are directly involved in neurodegenerative processes in SLE. Hippocampal brain abnormalities in NPSLE patients further support this notion (Appenzeller et al., 2006). Depending on the antibody concentrations, DNA–NMDA receptor antibodies can cause either neuronal dysfunction by transiently enhancing excitatory postsynaptic potentials or can result in neuronal cell death (Kowal et al., 2006; Faust et al., 2010). This evidence could be of high relevance in terms of reversibility of symptoms. Furthermore, anti-brain antibodies were also shown to induce neuropsychiatric symptoms in patients with other autoimmune disorders such as celiac disease (Alaedini et al., 2007; Hadjivassiliou et al., 2013) or inflammatory bowel diseases (Häuser et al., 2011; Papathanasiou et al., 2014). Taken together, anti-brain antibodies were shown to cause neuropsychiatric pathology in different diseases presenting novel therapeutic options.

These promising findings of anti-brain antibodies in systemic inflammatory disorders encouraged the search for anti-brain antibodies in inflammatory brain disorders. MS is characterized by oligoclonal bands in the CSF and antibodies in acute MS lesions (Lucchinetti et al., 2000). Histopathological studies confirm antibody mediated demyelination (Storch et al., 1998). Despite these findings all attempts to identify pathogenic antibodies to CNS antigens and infectious agents in the context of MS were rather unsatisfactory. In contrast to MS, highly specific anti-brain antibodies were discovered in NMO (Lennon et al., 2005), a disease which can closely resemble MS but requires different treatment. Antibodies to the water channel protein aquaporin-4 (AQP4) are detectable in around 80–90% of patients with NMO (Mader et al., 2010; Waters et al., 2012). AQP4 is localized on astrocytic endfeet forming the BBB. NMO is characterized by optic neuritis and longitudinal extensive transverse myelitis over three or more vertebral segments, which can lead to blindness and paralysis of patients (Wingerchuk et al., 1999; Cree, 2008). AQP4 antibody seropositivity is highly predictive for the disease (Matiello et al., 2008) and enables early treatment of patients. This is particularly important since IFN β, commonly used for treating MS, can worsen the disease outcome of NMO patients (Palace et al., 2010). Pathological findings show immunoglobulin and complement deposition around blood vessels with AQP4 specific loss in brain and spinal cord lesions (Lucchinetti et al., 2002). Rodents develop NMO-like lesions in the brain and spinal cord upon injection of patient derived AQP4 antibodies in an EAE animal model (Bennett et al., 2009; Bradl et al., 2009; Kinoshita et al., 2009). In addition, one study showed loss of astrocytes and demyelinating lesions after injection of AQP4 IgG into the brain together with human complement (Saadoun et al., 2010). AQP4 antibodies might require the help of encephalitogenic T-cells to breach the BBB (Saadoun et al., 2010; Pohl et al., 2013) and in addition CNS specific T-cells may require a local inflammatory environment for the antibodies at the lesion site (Pohl et al., 2013). Antibodies to AQP4 bind to astrocytes and lead to complement depend cytotoxicity as well as antibody dependent cytotoxicity, resulting in astrocytosis (Papadopoulos et al., 2014). Demyelination and neuronal cell death could occur as a secondary inflammatory response, due to secretion of toxic compounds such as nitrogen species, reactive oxygen or glutamate by activated astrocytes (Brosnan and Raine, 2013), which could lead to limited trophic support to myelin (Levy, 2014). Recently, cognitive impairment has been reported in NMO (Saji et al., 2013), yet this finding needs to be replicated in larger cohorts. The pathological role of AQP4 antibodies in cortical neuronal loss at areas of high AQP4 expression is not well understood. Although NMO is a rather rare disease, the findings obtained from AQP4 IgG and its contribution in glial injury, demyelination and neurodegeneration could be of direct relevance for MS and related inflammatory diseases, and will help to better understand the role of astrocytes in inflammation and neurodegeneration.

## Immune-Mediated Disruption of the Neurogenic Niche may Contribute to Neurodegeneration

A final mechanism potentially connecting systemic inflammation and neurodegeneration is impairment of neurogenesis. Neurogenesis is a central mechanism required for neuronal maintenance and adaptive plasticity in the healthy and diseased brain (Jin et al., 2006a). Inflammatory mediators have various effects on neurogenesis (Whitney et al., 2009). Impairment of neurogenesis was shown in neurodegenerative diseases such as AD (Lazarov and Marr, 2010) and neuropsychiatric disorders such as depression (Sahay and Hen, 2007). Interestingly, approved AD drugs (Jin et al., 2006b; Kotani et al., 2008) and chronic antidepressant treatment (Malberg et al., 2000; Santarelli et al., 2003) induce neurogenesis. Inflammation and microglial activation is detrimental for neurogenesis that can be restored by anti-inflammatory treatment (Ekdahl et al., 2003; Monje et al., 2003). Moreover, microglia are not only involved in the maintenance of the neurogenic niche (Sierra et al., 2010) but also in synaptic maintenance (Stevens et al., 2007; Parkhurst et al., 2013). Of interest, systemic immune cells were shown to be involved in regulation of neurogenesis. CD4^+^ T-cells were shown to promote (Wolf et al., 2009) while CD8^+^ T-cells impair proliferation of neural progenitor cells (Hu et al., 2014). An effect of B-cells on neurogenesis was not observed (Wolf et al., 2009). These findings need to be independently replicated. However, one may speculate that neuropsychiatric symptoms elicited by chronic inflammation may be driven by detrimental changes of neuronal homeostasis. Thus, specific immune modulatory treatment might be beneficial.

## Concluding Remarks

The immune and nervous systems have co-evolved from early invertebrates to higher mammals, creating an intricate bi-directional modulating dialog. The CAP is a good example of the influence of the nervous system upon the immune response. In the opposite direction, the activation of innate immunity in response to serious, as well as non-life threatening, infections induces the maturation and release of TNF, IL-1β, and other inflammatory cytokines that in turn cause transient anorexia, malaise, depression, and other features of the sickness syndrome (Figure 3). This is not surprising if we take into consideration that glia constitutes no less than half of the cells in a mammalian brain.

Sustained systemic inflammation is a common feature of many autoimmune disorders, and is present in most sepsis survivors. Cognitive impairment is common in sepsis survivors, as well as patients suffering from chronic inflammatory conditions. Cognition and behavior are persistently challenged, even after apparent resolution of acute sepsis. Moreover, systemic inflammation occurring in a susceptible brain (e.g., patients with AD) may lead to even further disruption in quality of life and activities of daily living. Up to 95% of patients with SLE develop neuropsychiatric dysfunction. In SLE, part of the repertoire of DNA-binding autoantibodies cross-react with hippocampal NMDA receptors, and – through a leaky BBB – gain access to the brain, inducing cognitive decline and other neuropsychiatric manifestations. In patients with rheumatoid arthritis, the baseline vagal tone of is persistently low, suggesting a possible mechanism for persistent inflammation. Those examples indicate that the normal neuroimmune cross-talk in health can become deleterious during disease, particularly in a primed brain – one with preexistent damage. Recently, cellular, molecular, environmental, and genetic components have been linked to the persistent brain disfunction of systemic inflammation. Here, we have discussed mechanistic evidence for the intricate interrelation between inflammation and neurodegeneration. Identification of druggable targets derived from these mechanisms holds the promise to prevent long-term disability and improve the quality of life in patients with chronic inflammatory conditions.

## Conflict of Interest Statement

The authors declare that the research was conducted in the absence of any commercial or financial relationships that could be construed as a potential conflict of interest.
